# A Novel Domperidone Hydrogel: Preparation, Characterization, Pharmacokinetic, and Pharmacodynamic Properties

**DOI:** 10.1155/2011/841054

**Published:** 2011-02-20

**Authors:** Chun-Hui Zhang, Bing-Xiang Zhao, Yue Huang, Ying Wang, Xi-Yu Ke, Bo-Jun Zhao, Xuan Zhang, Qiang Zhang

**Affiliations:** ^1^Department of Pharmaceutics, School of Pharmaceutical Sciences, Peking University, Beijing 100191, China; ^2^State Key Laboratory of Natural and Biomimetic Drugs, School of Pharmaceutical Sciences, Peking University, Beijing 100191, China

## Abstract

The purpose of the present study was to prepare a novel domperidone hydrogel. The domperidone dispersion was prepared by the solvent evaporation method. The characteristics of domperidone dispersion were measured by dynamic light scattering (DLS), scanning electronic microscopy (SEM), differential scanning calorimetry (DSC), X-ray diffractometry, and solubility test, respectively. Domperidone hydrogel was prepared by directly incorporating the domperidone dispersion in Carbopol hydrogel to increase its mucoadhesive properties to gastrointestinal tract (GIT). The *in vivo* pharmacokinetic and pharmacodynamic studies were investigated to evaluate the relative oral bioavailability and the propulsion efficacy of domperidone hydrogel as compared with market domperidone tablet (Motilium tablet). The particle size of domperidone dispersion in distilled water was 454.0 nm. The results of DSC and X-ray indicated that domperidone in dispersion was in amorphous state. The solubility of domperidone in the dispersion in distilled water, pH of 1, 5, and 7 buffer solution was 45.7-, 63.9-, 13.1-, and 3.7-fold higher than that of raw domperidone, respectively. The area under the plasma concentration curve (AUC_0–24_) in domperidone hydrogel was 2.2-fold higher than that of tablet. The prolonged propulsion efficacy in the domperidone hydrogel group compared to that in tablet group was observed in the pharmacodynamic test.

## 1. Introduction

Domperidone, a dopamine D_2_ receptor antagonist, is used as a prokinetic and antiemetic agent for the treatment of gastroparesis, nausea, and vomiting [1]. Domperidone is a weak base with good solubility in acidic pH but in alkaline pH, its solubility is significantly reduced [2]. The oral bioavailability of domperidone has been reported at the range of 13–17% [3]. The poor aqueous solubility may be one possible reason for its low bioavailability. In order to increase the bioavailability of domperidone, a controlled release dosage form has been prepared to increase the solubility of domperidone in the alkaline medium [2]. Recently, the domperidone gastric floating matrix tablet has been demonstrated to prolong the presence of the dosage form in the stomach or the upper small intestine and increase the amount of dissolved domperidone in gastric medium [4]. Therefore, it may play a key role of enhancing the solubility of domperidone in the aqueous medium. 

The oral bioavailability of amorphous drugs can be improved because of the increase of apparent solubility [5–7]. In addition, supersaturation of the drug in the gastrointestinal tract, particularly in the upper intestine, may lead to faster permeation rates through biomembranes and thus, enhance absorption [8–10]. Many polymers have been used as crystallization inhibitors to form amorphous solid dispersion in order to avoid forming lower energy crystalline states. These polymeric crystallization inhibitors orient preferentially to the water/drug particle interface to stabilize the particles. Recently, itraconazole amorphous nanoparticles and amorphous compositions have been reported to increase the supersaturation of itraconazole [11–19]. In addition, felodipine amorphous nanoparticles were investigated by an evaporation method using PVP as a hydrophilic polymeric carrier [20]. The author indicated that the interaction at the molecular level of drug with the polymer carrier would control the physical state and the particle size of drug-carrier system. Being controlled by the relatively strong interaction of felodipine with PVP, the drug forms amorphous particles in the nanometer size range. Otherwise, for felodipine/PEG system, the drug is dispersed as crystals having sizes in the microrange.

Mucoadhesive hydrogel prepared by bioadhesive polymer has the property of increasing the time of retention in GIT when it orally administrated. Carbopol is one of the currently most widely used mucoadhesive hydrogel polymers. Now, a relevant amount of work has been done on its bioadhesive properties [21–24]. In particular, Carbopols may be used in oral preparations to improve GIT retention time. It has been reported that the combination of nanosized drug particles with Carbopol hydrogel would increase mucoadhesive properties of the drug particles, and then achieve a sufficiently high bioavailability [25]. When nanosized drug particles are directly dispersed in the hydrogel, the mucoadhesive polymer will be absorbed onto the particle surface and the contact time of drug particles with GIT mucous membranes will further increase. 

The aim of the present study was to prepare a novel domperidone hydrogel and evaluate its *in vivo* properties. The domperidone dispersion was prepared by a solvent evaporation method using polyvinylpyrrolidone K30 as a hydrophilic polymer. The dynamic light scattering (DLS) technique was used to measure the particle size of the domperidone dispersion in distilled water. Morphological and thermal behaviors of the domperidone dispersion were examined by scanning electronic microscopy (SEM) and differential scanning calorimetry (DSC), respectively. Also, X-rays were used to investigate the characteristic of drug crystallinity. The solubility of domperidone in dispersion was tested. Domperidone hydrogel was prepared by directly incorporating the domperidone dispersion in Carbopol hydrogel. The *in vivo* pharmacokinetic and pharmacodynamic studies were investigated to evaluate the relative oral bioavailability and propulsion efficacy of domperidone hydrogel compared with market domperidone tablet (Motilium tablet).

## 2. Materials and Methods

### 2.1. Materials

Domperidone (the purity more than 99.5%) was purchased from the Baotai Pharmaceutical Co. of Shanxi, China. Polyvinylpyrrolidone K_30_ (PVP K_30_) was obtained from ISP Technologies, INC. (A local agent in Beijing, China). Carbopol 974 P was kindly provided by BF Goodrich Specialty Chemicals (a local agent in Beijing, China). The market domperidone tablet, Motilium tablet (10 mg/per tablet, Lot: 0611211114, Xian Janssen Co. China) was purchased from Beijing pharmacy. Absolute alcohol was obtained from the Beijing Chemical Factory. All other chemicals were of analytical grade or HPLC grade.

### 2.2. Animals

Healthy adult male beagle dogs weighting 9–11 kg and male Kunming mice weighing 18–21 g were supplied by the Department of Experimental Animals (Peking University Health Science Center) and maintained under natural light/dark conditions. Animals were acclimatized for 7 days prior to experiment and were allowed free access to standard food and water. Temperature and relative humidity were maintained at 25°C and 50%, respectively. All care and handling of animals were performed with the approval of Institutional Authority for Laboratory Animal Care of Peking University.

### 2.3. Preparation of Domperidone Dispersion and Physical Mixture

Domperidone dispersion was prepared by dissolving accurately weighed amounts of domperidone (50 mg) and PVP K_30_ (500 mg) (1 : 10 w/w) in dehydrated ethanol in a closed glass receiver. After complete dissolution, the solvent was evaporated under reduced pressure at 50°C to form a uniform film. Desiccation was completed in a vacuum oven until constant weight was achieved. After adding distilled water, the mixture was sonicated for five minutes and then lyophilized to form a pulverous domperidone dispersion using an FD-2B Lyophilizer (Boyikang Co., Beijing, China). 

Physical mixture was prepared by simple intensive mixing of domperidone and PVP K_30_ previously grinded for 1-2 min in a mortar until a homogeneous mixture was obtained. The resulting mixture was sieved through grade 60 and then stored in a desiccator at room temperature until use.

### 2.4. Particle Size Analysis

The particle size of domperidone dispersion in distilled water (1 mg domperidone dispersion suspended in 10 mL distilled water) was determined by dynamic light scattering (DLS) using a Zetasizer Nano-Instrument (Malvern Instruments, Nano ZS, ZEN3600, UK).

### 2.5. Scanning Electron Microscopy (SEM)

SEM analysis was carried out using a Jeol JSM-5600LV scanning electron microscope (Japan). Prior to examination, samples were gold sputter-coated to render them electrically conductive.

### 2.6. Differential Scanning Calorimetry (DSC)

The DSC studies were conducted using a Thermal Analysis DSC-Q100 differential scanning calorimeter (USA). About 5 mg of samples including raw domperidone, raw PVP, physical mixture, and domperidone dispersion were encapsulated in flat-bottomed aluminum pans. The thermograms were recorded at a heating rate of 10°C · min^−1^ from 30 to 280°C using nitrogen as the purging gas.

### 2.7. Powder X-Ray Diffraction (PXRD)

The powder X-ray diffraction patterns were obtained with a Rigaku Dmax/2400 apparatus (Japan) using Cu-K*α* radiation (*λ* = 1.541 nm), a voltage of 40 kV and a 100 mA current. Samples were scanned from 5–30°  2*θ* for qualitative studies and the scanning rate was 4° · min^−1^.

### 2.8. Solubility Determination

Excess amounts of raw domperidone or domperidone dispersion were added into 50 mL polypropylene conical tubes with suitable volume of buffer solution (pH 1.0, 5.0, and 7.0) or distilled water, respectively. Then, the capped tubes were agitated at 37°C in a thermostatically controlled water bath for 48 hours. After equilibrium had been attained, the solutions were immediately and rapidly filtered through a 0.22 *μ*m Millipore filter (supplied by Jingteng Science China Corp.) and the filtrate was diluted with buffer solutions or distilled water. The amount of domperidone in each diluted sample was analyzed by the HPLC system (Waters Co. Inc., Westerville, OH, USA), which was equipped with a 1525-pump, 2487-ultraviolet detector, and a Phenomenex ODS3 (250 × 4.60 mm, 5 *μ*m) chromatographic column. The mobile phase, composed of methanol-0.06 M ammonium acetate (70 : 30), was delivered at 1.0 mL/min. The injection volume was 20 *μ*L. The drug was detected at 287 nm and the retention time of the drug was ~10 min. The drug concentration in the filtrate represented its saturation solubility. The experiment was conducted in triplicate.

### 2.9. Preparation of Domperidone Hydrogel

Carbopol 974 P was dispersed in distilled water. After equilibrated for 24 h, the Carbopol 974 P suspension was neutralised by addition of triethanolamine (adjusted to pH 7.0–7.5) to obtain the hydrogel (0.25% w/w). The domperidone dispersion was directly incorporated into the hydrogel with stirring to obtain the domperidone hydrogel. The content of domperidone in hydrogel was 1mg per mL hydrogel.

### 2.10. Pharmacokinetic Studies of Domperidone Hydrogel in Beagle Dogs

Three adult male beagle dogs weighting 9–11 kg, after denied food overnight for at least 12 hours but had access to water ad libitum, were orally received marketed domperidone tablet or domperidone hydrogel at 8:00 a.m. with 30 mL of distilled water in a crossover manner with one week washout period, respectively. The dose of domperidone administered to each dog was 10 mg/body. All the experiments were carried out at the same time of the day to exclude the influence of circadian rhythms. After oral administration of domperidone formulations, blood sample (about 0.8 mL) was collected with glass vials containing lyophilized sodium heparin from the jugular vein at 0, 0.17, 0.33, 0.5, 1, 1.5, 2, 2.5, 3, 4, 6, 8, 12, 18, and 24 hours intervals. After centrifugation at 3000 g for 5 min, the plasma samples were obtained and stored at −20°C until analysis. The measurement method of domperidone in plasma was modified according to the previous report [26]. Briefly, an aliquot of 400 *μ*L plasma samples, 100 *μ*L propranolol solution (25 *μ*g/mL, as an internal standard, the purity was 99.22%), 100 *μ*L NaOH (0.1 M), 0.5 mL acetonitrile, and 4 mL absolute ether were mixed by a vortex mixer for 1 min. The mixture was centrifuged at 5000 g for 10 min. Then, a volume of 4.0 mL of supernatant was collected, and dried under a low flow of nitrogen gas at 50°C in a water bath. The residue was reconstituted using the mobile phase (100 *μ*L), then, an aliquot of this solution (50 *μ*L) was injected onto and assayed by Agilent 1100 HPLC system consisting of a G1321A spectrofluorometric detector (Agilent Co. Inc., USA). Mobile phase was consisted of methanol-0.02 M KH_2_PO_4_ (48 : 52, v/v), and delivered at a flow rate of 1 mL/min. Chromatographic separation was performed on a Phenomenex ODS_3_ column (250 × 4.6 mm, 5 *μ*m, Torrance, CA, USA), and maintained at 30°C by a column oven. The detector was set at 282 nm for excitation and 328 nm for emission wavelength. The peak area of domperidone (Ad) and propranolol (Ap) were recorded, and the concentration of domperidome was calculated according to the ratio of Ad/Ap. The limit of quantification (LOQ) of the assay was 1 ng/mL, and linearity was obtained for domperidone concentrations ranging from 5 to 100 ng/mL (*R* ^2^ = 0.9978). The coefficients of variation of the interday and intraday precision of the quality control samples ranged from 6.4% to 11.4% and accuracy ranged from 101 to 117%.

The pharmacokinetic parameters were calculated from the plasma levels by noncompartmental pharmacokinetic analysis using the software package WinNonLin v 5.2 (Pharsight Corporation, Mountain View, CA). The peak plasma concentration (*C*_max_) and time to reach peak plasma concentration (*T*_max_) was obtained from the visual inspection of the plasma concentration-time curves. The area under the plasma concentration curve (AUC_0-t_) was determined using the trapezoidal rule up to 24 hours after drug administration.

### 2.11. The Propulsion Efficacy of Domperidone Hydrogel in Mice

Kunming mice—after 12 hours of fasting—were divided into a Motilium tablet group and a domperidone hydrogel group (*n* = 8). Mice in this two domperidone preparation groups received Motilium tablet (the Motilium tablet was grinded and suspended in distilled water) or domperidone hydrogel by intragastric administration. The dose of domperidone administered to each animal was 5 mg/kg. After 0.5, 1.0, 1.50, 2.0, 2.5, 3.0, 4.0, and 6.0 h administrations, mice received 0.1 mL ink [27]. After 10 min, the mice were killed by cervical vertebral dislocation with their stomachs cut open to collect their intestines. The propulsion efficacy of domperidone in preparations was presented by ink propulsion rate. The ink propulsion rate was calculated by using the formula: ink propulsion rate % = migration distance of ink/the distance from pylorus-duodenum junction to ileocecum × 100%. The mice (*n* = 8) in control group intragastrically received 0.5 mL physiological saline. The ink propulsion rate was determined according to the procedures outlined above.

### 2.12. Statistics

Data was presented as the mean ± standard deviation (SD). One-way analysis of variance (ANOVA) was used to determine significance among groups, after which post hoc tests with the Bonferroni correction were used for comparisons between individual groups. Statistical significance was established at *P* < .05.

## 3. Results

### 3.1. Particle Size Analysis

**Figure 1:**
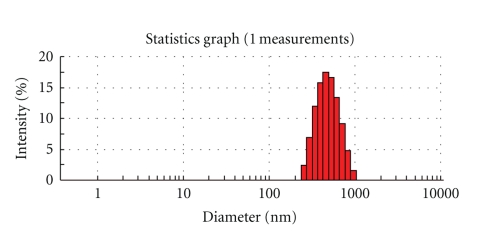
The particle size and distribution of domperidone dispersion. The particle size of domperidone dispersion in distilled water (1 mg domperidone dispersion suspended in 10 mL distilled water) was determined by dynamic light scattering (DLS).

The particle size of domperidone dispersion in distilled water was found to be 454.0 nm, as shown in Figure 1. The polydispersity index was 0.115.

### 3.2. Scanning Electron Microscopy (SEM)

**Figure 2:**
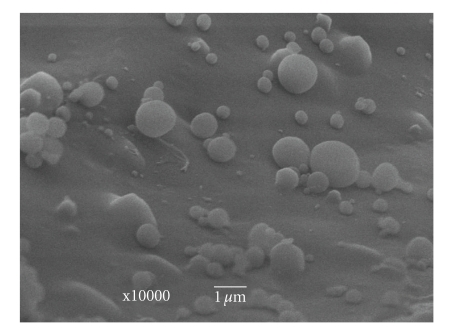
Scanning electron micrographs (SEM) photograph of domperidone dispersion.

The result of SEM imaging of domperidone dispersion, which is shown in Figure 2, indicated that the particles had nanometer-size spherical shapes with a rounded surface appearance and also that no drug crystal was visible.

### 3.3. Differential Scanning Calorimetry (DSC)

**Figure 3:**
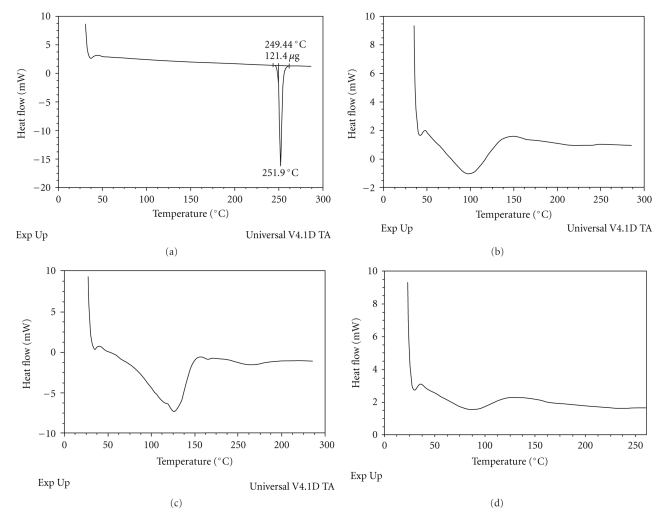
Differential scanning calorimetry (DSC) of raw domperidone (1), raw PVP K30 (2), physical mixture of domperidone and PVP K_30_ (3), and domperidone dispersion (4).

The DSC thermograms of raw domperidone, raw PVP, physical mixture, and domperidone dispersion are shown in Figure 3. The curve of raw domperidone showed an endothermic peak at 251.9°C. In case of the physical mixture, the endothermic peak of domperidone was broadened, shrunk, and shifted to 125°C. The complete disappearance of the drug endothermic peak was observed in the domperidone dispersion. This phenomenon can therefore assume that the domperidone in the dispersion was in an amorphous form.

### 3.4. Powder X-Ray Diffraction (PXRD)

**Figure 4:**
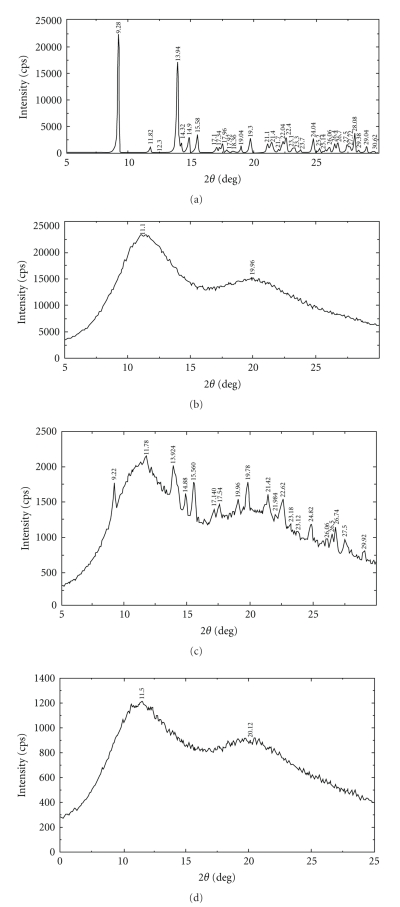
Powder X-ray diffractogram (PXRD) of raw domperidone (1); raw PVP K30 (2); physical mixture of domperidone and PVP K30 (3); domperidone dispersion (4).

The PXRD patterns for raw domperidone, raw PVP, domperidone dispersion, and the corresponding physical mixture are shown in Figure 4. In the X-ray diffraction spectrum, domperidone exhibited several strong characteristic crystalline peaks at 2*θ* = 9.28°, 13.94°, 15.58°, 19.80°, and 24.80° in raw domperidone, suggesting that the drug was present as a crystalline material. Some domperidone crystal peaks were still detected in the physical mixtures. In contrast, there were no sharp peaks attributable to the crystalline form in domperidone dispersion, suggesting that domperidone in this dispersion was in an amorphous state. This result confirmed the result obtained from DSC.

### 3.5. Solubility

The solubility of domperidone in raw domperidone and domperidone dispersion is presented in Table 1. In distilled water, the solubility of domperidone in the dispersion was 205.5 ± 3.7 *μ*g/mL, 45.7-fold higher than that of raw domperidone (4.5 ± 0.3 *μ*g/mL). In pH 1, 5, and 7 buffer solution, the solubility of domperidone in dispersion were 36211.8 ± 1.3, 3198.4 ± 21.7 and 10.6 ± 0.27 *μ*g/mL, 63.9-, 13.1- and 3.7-fold higher than that of raw domperidone (566.8 ± 50.9, 243.3 ± 4.2 and 2.9 ± 0.33 *μ*g/mL), respectively.

### 3.6. Pharmacokinetic Studies of Domperidone Hydrogel in Beagle Dogs

**Figure 5:**
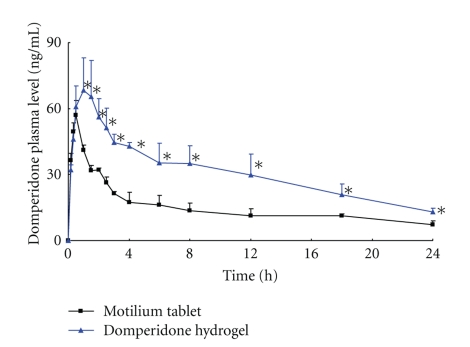
Concentration-time profiles of domperidone in plasma after a single oral administration of domperidone hydrogel or Motilium tablet to beagle dogs at a dose of 10 mg/body. Each point represents mean ± S.D. (*n* = 3). The asterisks indicate a statistically significant difference between domperidone hydrogel group and Motilium tablet group (*P* < .05).

The *in vivo* pharmacokinetic results obtained for the formulation based on domperidone hydrogel were compared with Motilium tablet.  Figure 5 showed the average plasma concentration versus time curves of domperidone after oral administration of preparations to beagle dogs at a dose of 10 mg/body. The concentrations of domperidone in hydrogel treatment group, especially in last time points, were remarkable higher than that in tablet treatment group (*P* < .05). 

As shown in Table 2, administration of Motilium tablet resulted in AUC_0-24_ values of 382.11 ± 52.71 h · ng/mL. When the same dose of domperidone was formulated in hydrogel, the systemic exposure to domperidone was raised significantly as reflected in an AUC_0–24_) of 829.64 ± 105.09 h · ng/mL higher than that from tablet (*P* < .01). In terms of *C*_max_, the values from hydrogel group (70.05 ± 12.27 ng/mL) were higher than those from tablet group (56.95 ± 6.63 ng/mL), however, there was no significant difference between these two groups. Based on a comparison of the *T*_max_ values, the *C*_max_ reached time in hydrogel group (1.17 ± 0.29 h) was significantly longer as compared to tablet group (0.44 ± 0.10 h) (*P* < .01). In addition, the differences in the values of Vz and Cl between hydrogel group and tablet group (*P* < .05 or *P* < .01) could also be noted. 

### 3.7. The Propulsion Efficacy of Domperidone Hydrogel in Mice

**Figure 6:**
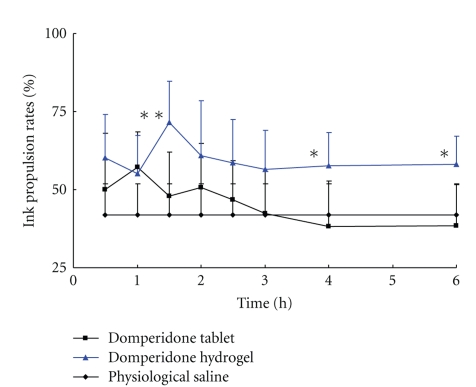
Profiles of the ink propulsion rates of the small intestine after intragastric administered Motilium tablet, domperidone hydrogel, or physiological saline to mice. Each point represents mean ± S.D. (*n* = 8). The dose of domperidone was 5 mg/kg. The asterisks indicate a statistically significant difference between domperidone hydrogel group and physiological saline group (*P < .05; **P < .01).

Figure 6 shows the ink propulsion rates of small intestine in mice after they were administered intragastric Motilium tablet, domperidone hydrogel, or physiological saline. The ink propulsion rates in domperidone preparation groups at peak time were significantly higher than that in tablet or physiological saline group (*P* < .05 or *P* < .01), as shown in Table 3. The difference in ink propulsion rates at peak time point between the hydrogel group and tablet group were significant (*P* < .05). The values of ink propulsion rates at 6 h time point in hydrogel group compared with the tablet or physiological saline group were significant higher (*P* < .05), whereas there was no significant difference between the physiological saline group and tablet group (Table 3).

The peak time of ink propulsion rates in domperidone hydrogel group and tablet group was 1.5 and 1 h, respectively. In fact, the values of ink propulsion rates in domperidone hydrogel group at 0.5 h time points, (60.1 ± 14.0)%, was similar with those in tablets group in 1 h time point (at peak time point, (57.1 ± 11.5)%), indicating that the onset time of propulsion efficacy in hydrogel group was faster than that in tablet group. The propulsion efficacy in the domperidone hydrogel group was sustained at least 6 h; however, it was sustained only 3 h in tablet group, indicating the prolonged propulsion efficacy in hydrogel group compared to that in tablet group.

## 4. Discussion

In the present study, we prepared domperidone dispersion by evaporation method using PVP as a polymeric crystallization inhibitor. The amorphous domperidone in dispersion was confirmed by DSC and X-ray tests. The particle size of domperidone dispersion in distilled water and the morphology of domperidone dispersion indicated that the domperidone dispersion was in the nanometer size range. The solubility of domperidone in the dispersion in distilled water was found to be 45.7-fold higher than that of raw domperidone. Similar results were also found in pH 1 and 5 buffer solution, 63.9- and 13.1-fold higher than that of raw domperidone. The higher solubility of domperidone in the dispersion would provide a guarantee of enhancing the absorption of domperidone in GIT.

The solubility of domperidone in dispersion in pH 7.0 buffer solution was 10.6 *μ*g/mL. According to the calculation, about 99.9% domperidone dispersion existed as solid nanosized particles in this solution. Therefore, we suggest that almost all of the domperidone dispersion in the Carbopol hydrogels would exist as solid nanosized particles. 

Carbopol is a very useful polymer for hydrogel delivery system [28, 29]. The incorporation of nanoparticles with hydrogel would increase its adhesive properties to GIT. This is a benefit for enhancing drug oral bioavailability. In addition, Carbopol hydrogel has the characteristic of a higher viscosity, which could limit the sedimentation of the contained particles. Excellent long-term stability of the hydrogel/drug nanoparticles system has been observed by Müller and Jacobs [25]. Even so, the stability of domperidone dispersion existed as nanosize particles in the Carbopol hydrogel should be further investigate in future. 

The physical properties of the Carbopol hydrogels are extremely sensitive to the presence and concentration of additives [30]. It has been reported that for Carbopol hydrogels with 0.05 and 0.1% of PVP, the behavior is similar to that of single Carbopol formulation, their pseudoplastic non-Newtonian character being maintained [21]. Therefore, in the present research, the addition of PVP does not substantially modify the rheological behavior of the Carbopol hydrogels. 

Considering the advantages of amorphous dispersion and adhesive properties of hydrogel to the GIT wall, we incorporated domperidone dispersion with Carbopol hydrogel to prepare a novel domperidone hydrogel. The *in vivo* pharmacokinetic evaluation of domperidone hydrogel in dogs was investigated. The positive pharmacokinetic results showed that the AUC in domperidone hydrogel was 2.2-fold higher than that in Motilium tablet. The concentrations of domperidone in hydrogel treatment group, especially in last time points, were remarkable higher than that in tablet treatment group (*P* < .01). The results of *in vivo* pharmacodynamic evaluation in mice indicated that the higher and prolonged propulsion efficacy was observed in the domperidone hydrogel group compared to that in tablet group. We suggested that the particle size of domperidone reduction to a nanometer range and the conversion of crystalline drug to amorphous state in dispersion resulted in an increase of dissolution velocity and degree of the drug in the GIT, especially in gastric fluid. Therefore, the rapid and complete absorption from the gastrointestinal tract would lead to improving the bioavailability and the propulsion efficacy of domperidone in hydrogel group. Another reason for the *in vivo* results was the role of Carbopol hydrogel which prolonged the residence and the contact time of domperidone in GIT. The sustained release behavior in domperidone hydrogel group was confirmed by our pharmacokinetic and pharmacodynamic results.

## 5. Conclusion

The characterization of domperidone dispersion demonstrated that the prepared domperidone dispersion were amorphous and in the nanometer size range. Solubility of domperidone in the dispersion showed a marked increase. The domperidone hydrogel was prepared by directly incorporating the domperidone dispersion in Carbopol hydrogel. As a result of the mucoadhesive properties and improved solubility, a statistically significant improvement in bioavailability and prolonged propulsion efficacy of domperidone in hydrogel group were observed compared to that in Motilium tablet group in beagle dogs and mice. In addition, these results indicate that dispersion incorporating with hydrogel can be an effective tool to improve the bioavailability of poor water soluble drugs.

## Figures and Tables

**Table 1:** Solubility of domperidone in buffer solutions and distilled water (mean ± SD, *n* = 3). The drug concentration (*μ*g/mL) represents its solubility.

pH	Raw domperidone	Domperidone dispersion
1.0	566.8 ± 50.9	36211.8 ± 1.3
5.0	243.3 ± 4.2	3198.4 ± 21.7
7.0	2.9 ± 0.33	10.6 ± 0.27
Distilled water	4.5 ± 0.3	205.5 ± 3.7

**Table 2:** Selected pharmacokinetic parameters of domperidone after a single administration of Motilium tablet or domperidone hydrogel to beagle dogs at a dose of 10 mg/body (*n* = 3).

Parameter	Units	Motilium tablet	Domperidone hydrogel
Tmax	h	0.44 ± 0.10	1.17 ± 0.29**
Cmax	ng/mL	56.95 ± 6.63	70.05 ± 12.27
AUC_0-24_	H · ng/mL	382.11 ± 52.71	829.64 ± 105.09**
Vz	l/kg	35.16 ± 2.06	17.49 ± 2.17*
Cl	l/h/kg	2.04 ± 0.26	0.93 ± 0.08**
MRT	h	10.04 ± 1.05	10.85 ± 0.49

*P < .05 or **P < .01 versus tablet group.

**Table 3:** The ink propulsion rate of the small intestine at peak time or 6 h time point after an intragastric given Motilium tablet or domperidone hydrogel to mice at a dose of 5 mg/kg and the average ink propulsion rate of the small intestine after intragastric given physiological saline to mice. Each point represents mean ± S.D. (*n* = 8).

Ink propulsion rate (%)	Average	Peak time	6 h time point
Physiological saline group	41.9 ± 9.9	/	/
Motilium tablet group	/	57.1 ± 11.5*	38.3 ± 13.2
Domperidone hydrogel group	/	71.5 ± 13.3^∗∗∆^	58.0 ± 9.1^∗∆^

*P < .05, **P < .01 versus physiological saline group.

^∆^ *P* < .05 versus tablet group.
